# Predictive model for diabetic retinopathy under limited medical resources: A multicenter diagnostic study

**DOI:** 10.3389/fendo.2022.1099302

**Published:** 2023-01-05

**Authors:** Yanzhi Yang, Juntao Tan, Yuxin He, Huanhuan Huang, Tingting Wang, Jun Gong, Yunyu Liu, Qin Zhang, Xiaomei Xu

**Affiliations:** ^1^ Department of Endocrinology and Metabolism, Chengdu First People’s Hospital, Chengdu, China; ^2^ Operation Management Office, Affiliated Banan Hospital of Chongqing Medical University, Chongqing, China; ^3^ Department of Medical Administration, Affiliated Banan Hospital of Chongqing Medical University, Chongqing, China; ^4^ Department of Nursing, the First Affiliated Hospital of Chongqing Medical University, Chongqing, China; ^5^ College of Medical Informatics, Chongqing Medical University, Chongqing, China; ^6^ Department of Information Center, The University Town Hospital of Chongqing Medical University, Chongqing, China; ^7^ Medical Records Department, the Second Affiliated Hospital of Chongqing Medical University, Chongqing, China; ^8^ Department of Infectious Diseases, The First Affiliated Hospital of Chongqing Medical University, Chongqing, China; ^9^ Department of Gastroenterology, Chengdu Fifth People’s hospital, Chengdu, China

**Keywords:** diabetes mellitus, diabetic retinopathy, predictive model, medically underserved settings, webpage

## Abstract

**Background:**

Comprehensive eye examinations for diabetic retinopathy is poorly implemented in medically underserved areas. There is a critical need for a widely available and economical tool to aid patient selection for priority retinal screening. We investigated the possibility of a predictive model for retinopathy identification using simple parameters.

**Methods:**

Clinical data were retrospectively collected from 4, 159 patients with diabetes admitted to five tertiary hospitals. Independent predictors were identified by univariate analysis and least absolute shrinkage and selection operator (LASSO) regression, and a nomogram was developed based on a multivariate logistic regression model. The validity and clinical practicality of this nomogram were assessed using concordance index (C-index), area under the receiver operating characteristic curve (AUROC), calibration curves, decision curve analysis (DCA), and clinical impact curves (CIC).

**Results:**

The predictive factors in the multivariate model included the duration of diabetes, history of hypertension, and cardiovascular disease. The three-variable model displayed medium prediction ability with an AUROC of 0.722 (95%CI 0.696-0.748) in the training set, 0.715 (95%CI 0.670-0.754) in the internal set, and 0.703 (95%CI 0.552-0.853) in the external dataset. DCA showed that the threshold probability of DR in diabetic patients was 17-55% according to the nomogram, and CIC also showed that the nomogram could be applied clinically if the risk threshold exceeded 30%. An operation interface on a webpage (https://cqmuxss.shinyapps.io/dr_tjj/) was built to improve the clinical utility of the nomogram.

**Conclusions:**

The predictive model developed based on a minimal amount of clinical data available to diabetic patients with restricted medical resources could help primary healthcare practitioners promptly identify potential retinopathy.

## 1 Introduction

In China, the number of people with diabetes mellitus (DM) has increased significantly over the last four decades. The prevalence of DM has increased more than 15-fold, from 0.67% (1) in 1980 to 11.2% (2)in recent population studies. Diabetic retinopathy (DR) is a common chronic complication of DM and a leading cause of irreversible visual impairment in working-age adults (3).

The fact that patients with DR (including diabetic macular edema) may be asymptomatic in the early stages provides strong evidence for conventional retinal screening. The guidelines recommend that patients with type 1 or type 2 diabetes should undergo an initial comprehensive eye examination within 5 years after the onset of diabetes or at the time of diagnosis, respectively (4, 5). However, the capacity for early DR testing is insufficient, and there are few national programs for DR screening in China (6),especially given the high incidence of DM. The causes are numerous and include a lack of effective screening tools, scarcity of eye care professionals, and a multidisciplinary strategy from picture capture to DR diagnosis. In addition, regional economic imbalances and disparities in lifestyle make it challenging to replicate the DR management approaches in China. This is particularly problematic in restricted medical resource settings, where the annual screening rates for DR are significantly lower than the national average (7, 8).

Several molecular and biochemical pathways are involved in the incidence and development of DR, but the interactions between various mechanisms remain to be fully elucidated (9). Clinical studies have identified a number of risk factors for DR, including demographic characteristics such as age (10–12), duration of diabetes (10, 11, 13–17), obesity (10), and pregnancy status in diabetic women of childbearing age (18), comorbidities or complications such as hypertension (10, 12, 16), dyslipidemia (10, 12, 13), cardiovascular disease(CVD) (11, 12) and diabetic nephropathy (10, 12, 17, 19), and other laboratory parameters such as glycated hemoglobin (10, 11, 13, 15–17, 19), glycemic variability (20, 21), and susceptibility genes (22). However, the aforementioned risk factors derived from population-based studies can only account for 9% of DR progression (23). Laboratory biomarkers are difficult to obtain in healthcare resource-limited settings, and the rate of self-monitoring of blood glucose (SMBG) adherence in Chinese diabetic individuals has not reached an optimal level (24).

Considerable research effort has recently focused on developing predictive models for DR using machine learning algorithms (10, 12, 13, 16, 19, 21), but these models cannot be applied well in low-medical-resource settings. Nearly 510 million Chinese people (36.1% of the total population) live in rural areas (25), and 72.5% of patients do not comply with structured SMBG (24), not to mention consecutive follow-up records or regular laboratory tests. Medical providers in this primary health care (PHC) setting require a simple screening tool for quickly identifying patients at high risk of DR in a single visit and appropriately referring them to retinal specialist ophthalmologists.

This study aimed to provide a direct diagnostic paradigm for PHC clinicians and to develop an online application based on the nomogram so that patients at risk of DR without access to routine eye examinations could be prioritized for retinal screening.

## 2 Methods

### 2.1 Data source

Clinical data were obtained from five tertiary hospitals in southwest China, including five in Chongqing (hospitals A-D) and one in Chengdu (hospital E). In this multicenter retrospective study, 4,159 of 32,168 diabetic patients with clinical consultation records met the quality standards for the final analysis. These patients were randomly divided into a training set with 2,610 samples and an internal validation set with 1,119 samples from hospitals A-D. A total of 430 samples from hospital E were used for external validation. The study adhered to the principles of the Declaration of Helsinki and the Transparent Reporting of a Multivariable Prediction Model for Individual Prognosis or Diagnosis Guidelines (26). Clinical research ethics approval was obtained from the Ethics Committee of the Affiliated Banan Hospital of Chongqing Medical University. Individual patient-level consent was not required because the study only used fully de-identified collected data.

### 2.2 Diagnostic criteria

DM was diagnosed according to the 1999 World Health Organization criteria, consistent with the standards of medical care for type 2 diabetes in China (2019) (5). According to these guidelines (4, 5), the diagnosis of DR was determined based on fundus photography, examination using dilated funduscopy on ophthalmologist consultation, or prior medical records. Clinical diagnosis (CVD, hypertension, DR) and symptoms in our database are recorded using the ICD-10 code system. ICD-10 codes related to our study are given in **Table S1**.

### 2.3 Inclusion and exclusion criteria

The inclusion criteria for this study were diabetes patients admitted between July 2010 and June 2022 with laboratory parameters and ocular variables. Exclusion criteria were as follows: (i) age <18 years, (ii) data on diabetes duration and body matrix index (BMI) not available, and (iii) variables with >30% missing values. The detailed selection process is shown in **Supplementary Figure S1**.

### 2.4 Data collection

On the basis of previous studies, 42 variables routinely tested or recorded were collected, which included age, duration of diabetes, body mass index (BMI), systolic blood pressure (SBP), diastolic blood pressure (DBP), gender, smoking and alcohol consumption status, previous diagnosis of hypertension, cardiovascular disease (CVD) and stroke, antihypertensive drug therapy, lipid-lowering treatment (statins, fenofibrate), antiplatelet therapy, antidiabetic drugs [insulin, thiazolidinediones (TZD), alpha-glucosidase inhibitors (AGI), sulfonylurea (SU), dipeptidyl peptidase-4 inhibitor (DPP-4i), GLP-1 receptor agonists (GLP-1RAs), metformin (Met), sodium-glucose cotransporter-2 inhibitors (SGLT2-i)], glycated hemoglobin (HbA_1_c), fasting blood glucose (FBG), estimated glomerular filtration rate (eGFR), serum levels of creatinine (SCR), uric acid (SUA), albumin (ALB), total bilirubin (TBIL), alkaline phosphatase (AKP), aspartate aminotransferase (AST), alanine aminotransferase (ALT), total cholesterol (TC), triglycerides (TG), low-density lipoprotein cholesterol (LDL-C), neutrophil percentage (NP), neutrophil-to-lymphocyte ratio (NLR), platelet-to-lymphocyte ratio (PLR), lymphocyte to monocyte ratio (LMR), Systemic immune-inflammation index (SII; platelet count × neutrophil-to-lymphocyte ratio), and neutrophil percentage-to-albumin ratio (NPAR). eGFR was calculated using equations from the Modification of Diet in Renal Disease (MDRD), according to current recommendations (5). NLR, PLR, LMR, SII, and NPAR have been used to evaluate systemic inflammation, and these novel markers were recently found to be related to DR based on results from the National Health and Nutrition Examination Survey (NHANES) (27–29).

### 2.5 Statistical analysis

Statistical analysis was performed using SPSS 22.0 and R software (version 4.0.2, Vienna, Austria). Normally distributed variables were expressed as the mean ± standard deviation and compared by t-test between two groups. The median (interquartile range, [IQR]) was used to express variables without normal distributions, and the Mann-Whitney U test was used to compare them. Qualitative data were reported as percentages and compared using the χ^2^ test. Independent risk factors were selected using least absolute shrinkage and selection operator (LASSO) regression and multivariate logistic regression (30). A nomogram for identifying DR occurrence was constructed using covariates selected by multivariate regression, and discriminatory ability was assessed by measuring the area under the receiver operating characteristic curve (AUROC) (31). Calibration curves were used to evaluate calibration of the nomogram (32). Decision curve analysis and clinical impact curves were used to assess the clinical applicability of the nomogram (33). Multiple interpolation was used to fill in missing values that did not exceed 30% for continuous variables. All statistical analyses were two-sided, and statistical significance was set at P < 0.05.

## 3 Results

### 3.1 Patient characteristics

**Table 1** summarizes the clinical characteristics of patients in the training and internal validation sets. The two groups did not show significant differences in age, sex, history of smoking or alcohol consumption, hypertension, CVD, stroke, antihypertensive drugs, lipid-lowering treatment (statins, fenofibrate), antiplatelet therapy, antidiabetic drugs (insulin, TZD, AGI, SU, DPP-4i, GLP-1 Ras, Met, SGLT2-i), duration, BMI, SBP, DBP, or most laboratory parameters (P > 0.05).

**Table 1:** Demographic and clinical characteristics of the training and internal validation sets.

Variables	Training set (n = 2610)	Internal validation set (n = 1119)	P value
Age (IQR, years)	63.00 (54.00,70.00)	64.00 (54.00,71.50)	0.563
Sex			0.923
Female	1317 (50.46)	562 (50.22)	
Male	1293 (49.54)	557 (49.78)	
Smoking (yes, n, %)	819 (31.38)	346 (30.92)	0.811
Alcohol (yes, n, %)	706 (27.05)	287 (25.65)	0.397
DR (yes, n, %)	455 (17.43)	169 (15.10)	0.087
Hypertension (yes, n,%)	1005 (38.51)	396 (35.39)	0.078
CVD (yes, n, %)	363 (13.91)	141 (12.60)	0.309
Stroke (yes, n, %)	176 (6.74)	64 (5.72)	0.274
Antihypertensive drug (yes, n, %)	1260 (48.28)	519 (46.38)	0.305
Statins (yes, n, %)	1345 (51.53)	592 (52.90)	0.464
Fenofibrate (yes, n, %)	190 (7.28)	75 (6.70)	0.576
Antiplatelet drug (yes, n, %)	1493 (57.20)	622 (55.59)	0.380
Antidiabetic drug			
Insulin (yes, n, %)	2003 (76.74)	864 (77.21)	0.788
TZD (yes, n, %)	117 (4.48)	52 (4.65)	0.893
AGI (yes, n, %)	872 (33.41)	341 (30.47)	0.086
SU (yes, n, %)	985 (37.74)	423 (37.80)	1.000
DPP-4i (yes, n, %)	57 (2.18)	31 (2.77)	0.335
GLP-1RAs (yes, n, %)	74 (2.84)	31 (2.77)	0.999
Met (yes, n, %)	952 (36.48)	425 (37.98)	0.403
SGLT2-i (yes, n, %)	115 (4.41)	60 (5.36)	0.238
Duration (IQR, years)	9.00 (4.00,14.00)	8.00 (4.00,13.00)	0.128
BMI (IQR, kg/m^2^)	24.20 (22.00,26.70)	24.20 (21.70,26.70)	0.495
SBP (IQR, mmHg)	136.00 (124.00,150.00)	136.00 (123.50,149.00)	0.953
DBP (IQR, mmHg)	79.00 (71.00,87.00)	79.00 (70.00,86.00)	0.408
NP (IQR)	64.50 (57.18,72.30)	64.00 (57.25,71.95)	0.773
NLR (IQR)	2.39 (1.70,3.62)	2.31 (1.70,3.49)	0.610
PLR (IQR)	110.89 (83.71,150.00)	110.24 (81.70,150.00)	0.523
LMR (IQR)	4.62 (3.30,6.37)	4.80 (3.42,6.41)	0.224
SII (IQR)	435.30 (288.25,704.11)	430.61 (276.31,687.38)	0.475
NPAR (IQR)	15.60 (13.50,18.20)	15.50 (13.60,18.10)	0.814
TBIL (IQR, umol/l)	10.70 (8.10,14.30)	10.60 (8.00,14.20)	0.622
ALP (IQR, IU/L)	77.00 (63.00,95.53)	78.00 (63.00,95.00)	0.699
AST (IQR, IU/L)	20.00 (16.00,26.00)	19.00 (16.00,24.90)	0.023
ALT (IQR, IU/L)	19.65 (14.00,29.73)	19.00 (13.40,27.70)	0.008
TC (IQR, mmol/l)	4.58 (3.85,5.34)	4.59 (3.80,5.43)	0.314
TG (IQR, mmol/l)	1.55 (1.06,2.38)	1.57 (1.10,2.29)	0.686
LDL-C (IQR, mmol/L)	2.56 (1.97,3.19)	2.61 (2.01,3.25)	0.217
Cr (IQR, umol/l)	63.40 (51.70,79.20)	64.10 (52.15,81.10)	0.254
URAC (IQR, umol/l)	315.25 (255.78,381.33)	311.10 (257.30,387.30)	0.991
Alb (IQR,g/L)	41.10 (38.10,43.90)	40.90 (37.90,44.05)	0.990
HbA_1_c (IQR, %)	8.90 (7.40,11.10)	9.10 (7.40,11.40)	0.258
FBG (IQR, mmol/l)	9.61 (6.81,14.30)	9.50 (6.69,13.98)	0.396
eGFR (IQR, ml/min/1.73m^2^)	99.45 (79.74,116.89)	97.87 (77.95,116.20)	0.302

DR, diabetic retinopathy; CVD, cardiovascular disease; TZD, thiazolidinediones; AGI, alpha-glucosidase inhibitors; SU, sulfonylurea; DPP-4i, dipeptidyl peptidase-4 inhibitor; GLP-1RAs, GLP-1 receptor agonists; Met, metformin; SGLT2-i, sodium-glucose cotransporter-2 inhibitors; BMI, body mass index; SBP, systolic blood pressure; DBP, diastolic blood pressure; NP, neutrophil percentage; NLR, neutrophil-to-lymphocyte ratio; PLR, platelet-to-lymphocyte ratio; LMR, lymphocyte to monocyte ratio; SII, systemic immune-inflammation index; NPAR, neutrophil percentage-to-albumin ratio; TBIL, total bilirubin; ALP, alkaline phosphatase; AST, aspartate aminotransferase; ALT, alanine aminotransferase; TC, total cholesterol; TG, triglycerides; LDL-C, low-density lipoprotein cholesterol; Cr, serum levels of creatinine; URAC, uric acid; Alb, albumin; HbA1c, glycated hemoglobin; FBG, fasting blood glucose; eGFR, estimated glomerular filtration rate; IQR, interquartile range.

### 3.2 Selection of predictors for DR in the training set

Patients in the training set were divided into DR and non-DR groups. Univariate analysis revealed that the following variables were significantly associated with DR: age, sex, hypertension, CVD, stroke, antihypertensive drugs, statins, antiplatelet drugs, antidiabetic drugs (insulin, TZD, AGI, DPP-4i, GLP-1 RAs, Met), duration, BMI, SBP, SII, TBIL, TC, Cr, LDL-C, Alb, HbA1c, FBG, and eGFR **(** **Table 2** **)**.

**Table 2:** Univariate analysis of variables associated with DR.

Variables	Total (N=2610)	DR group (N=455)	Non-DR group (N=2155)	P value
Age	63.00 (54.00,70.00)	65.00 (57.00,71.50)	63.00 (53.00,70.00)	<0.001
Sex				0.031
Female	1317 (50.46)	251 (55.16)	1066 (49.47)	
Male	1293 (49.54)	204 (44.84)	1089 (50.53)	
Smoking (yes, n, %)	819 (31.38)	152 (33.41)	667 (30.95)	0.332
Alcohol (yes, n, %)	706 (27.05)	129 (28.35)	577 (26.77)	0.529
Hypertension (yes, n, %)	1005 (38.51)	286 (62.86)	719 (33.36)	<0.001
CVD (yes, n, %)	363 (13.91)	128 (28.13)	235 (10.90)	<0.001
Stroke (yes, n, %)	176 (6.74)	55 (12.09)	121 (5.61)	<0.001
Antihypertensive drug (yes, n, %)	1260 (48.28)	291 (63.96)	969 (44.97)	<0.001
Statins (yes, n, %)	1345 (51.53)	280 (61.54)	1065 (49.42)	<0.001
Fenofibrate (yes, n, %)	190 (7.28)	30 (6.59)	160 (7.42)	0.602
Antiplatelet drug (yes, n, %)	1493 (57.20)	331 (72.75)	1162 (53.92)	<0.001
Antidiabetic drug				
Insulin (yes, n, %)	2003 (76.74)	377 (82.86)	1626 (75.45)	0.001
TZD (yes, n, %)	117 (4.48)	35 (7.69)	82 (3.81)	<0.001
AGI (yes, n, %)	872 (33.41)	186 (40.88)	686 (31.83)	<0.001
SU (yes, n, %)	985 (37.74)	170 (37.36)	815 (37.82)	0.897
DPP4-i (yes, n, %)	57 (2.18)	19 (4.18)	38 (1.76)	0.003
GLP-1 RAs (yes, n, %)	74 (2.84)	24 (5.27)	50 (2.32)	0.001
Met (yes, n, %)	952 (36.48)	186 (40.88)	766 (35.55)	0.036
SGLT2-i (yes, n, %)	115 (4.41)	22 (4.84)	93 (4.32)	0.715
Duration (IQR, years)	9.00 (4.00,14.00)	12.00 (7.50,20.00)	8.00 (4.00,12.00)	<0.001
BMI (IQR, kg/m^2^)	24.20 (22.00,26.70)	24.70 (22.40,27.20)	24.20 (21.80,26.60)	<0.001
SBP (IQR, mmHg)	136.00 (124.00,150.00)	138.00 (126.00,152.00)	135.00 (123.00,148.00)	0.015
DBP (IQR, mmHg)	79.00 (71.00,87.00)	78.00 (71.00,86.00)	79.00 (72.00,87.00)	0.213
NP (IQR)	64.50 (57.18,72.30)	64.30 (56.80,70.60)	64.50 (57.25,72.50)	0.280
NLR (IQR)	2.39 (1.70,3.62)	2.39 (1.71,3.36)	2.40 (1.70,3.68)	0.636
PLR (IQR)	110.89 (83.71,150.00)	107.00 (84.30,143.44)	111.45 (83.50,150.59)	0.191
LMR (IQR)	4.62 (3.30,6.37)	4.43 (3.40,5.97)	4.66 (3.27,6.46)	0.230
SII (IQR)	435.30 (288.25,704.11)	422.38 (278.28,644.7)	440.53 (290.38,724.03)	0.048
NPAR (IQR)	15.60 (13.50,18.20)	15.70 (13.80,18.20)	15.50 (13.50,18.10)	0.234
TBIL (IQR, umol/l)	10.70 (8.10,14.30)	9.60 (7.50,12.50)	10.90 (8.30,14.70)	<0.001
ALP (IQR, IU/L)	77.00 (63.00,95.53)	75.00 (63.00,93.00)	78.00 (63.60,96.15)	0.056
AST (IQR, IU/L)	20.00 (16.00,26.00)	20.00 (16.00,26.00)	20.00 (16.00,26.00)	0.556
ALT (IQR, IU/L)	19.65 (14.00,29.73)	21.00 (14.55,30.00)	19.10 (14.00,29.50)	0.211
TC (IQR, mmol/l)	4.58 (3.85,5.34)	4.29 (3.61,4.97)	4.63 (3.90,5.43)	<0.001
TG (IQR, mmol/l)	1.55 (1.06,2.38)	1.49 (1.03,2.20)	1.56 (1.07,2.42)	0.087
LDL-C (IQR, mmol/L)	2.56 (1.97,3.19)	2.29 (1.77,2.90)	2.61 (2.00,3.25)	<0.001
Cr (IQR, umol/l)	63.40 (51.70,79.20)	66.20 (52.60,84.25)	63.00 (51.40,78.15)	0.009
URAC (IQR, umol/l)	315.25 (255.78,381.33)	323.80 (264.95,387.65)	313.40 (254.45,379.30)	0.056
Alb (IQR,g/L)	41.10 (38.10,43.90)	40.30 (37.60,43.00)	41.20 (38.30,44.10)	<0.001
HbA_1_c (IQR, %)	8.90 (7.40,11.10)	8.50 (7.30,10.50)	9.00 (7.40,11.20)	0.008
FBG (IQR, mmol/l)	9.61 (6.81,14.30)	9.07 (6.62,13.29)	9.77 (6.85,14.48)	0.026
eGFR (IQR, ml/min/1.73m^2^)	99.45 (79.74,116.89)	93.92 (75.60,107.16)	100.89 (81.91,118.77)	<0.001

DR, diabetic retinopathy; CVD, cardiovascular disease; TZD, thiazolidinediones; AGI, alpha-glucosidase inhibitors; SU, sulfonylurea; DPP-4i, dipeptidyl peptidase-4 inhibitor; GLP-1RAs, GLP-1 receptor agonists; Met, metformin; SGLT2-i, sodium-glucose cotransporter-2 inhibitors; BMI, body mass index; SBP, systolic blood pressure; DBP, diastolic blood pressure; NP, neutrophil percentage; NLR, neutrophil-to-lymphocyte ratio; PLR, platelet-to-lymphocyte ratio; LMR, lymphocyte to monocyte ratio; SII, systemic immune-inflammation index; NPAR, neutrophil percentage-to-albumin ratio; TBIL, total bilirubin; ALP, alkaline phosphatase; AST, aspartate aminotransferase; ALT, alanine aminotransferase; TC, total cholesterol; TG, triglycerides; LDL-C, low-density lipoprotein cholesterol; Cr, serum levels of creatinine; URAC, uric acid; Alb, albumin; HbA1c, glycated hemoglobin; FBG, fasting blood glucose; eGFR, estimated glomerular filtration rate; IQR, interquartile range.

**Figure 1:**
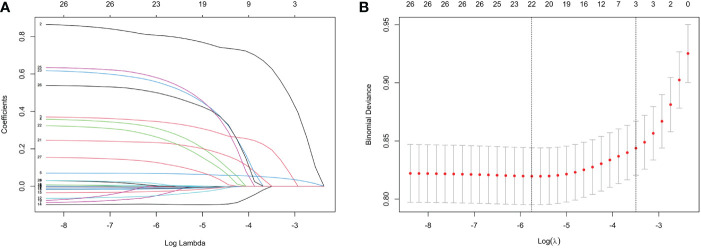
Features selection by LASSO. **(A)** LASSO coefficients profiles (y-axis) of the 26 features. The upper x-axis is the average numbers of predictors and the lower x-axis is the log(λ). **(B)** 10-fold cross-validation for tuning parameter selection in the LASSO model.

Indicators with statistical differences in univariate analysis were further by LASSO regression and multivariate logistic regression analysis (**Figure 1**, **Table 3**). In LASSO regression, ten-fold crossover method was used to verify the adjusted parameters λ. **Figure 1** shows the process of LASSO screening for optimal parameters. In the solution path diagram of λ and variables, as λ keeps increasing, the impact of the shrinkage penalty grows, the compression of the model gets stronger, and the fewer variables are selected. Each line represents the change of coefficients of different variables, and the different number of predictors selected according to λ and the coefficients corresponding to the selected variables are shown in **Figure 1A**. **Figure 1B** shows the logarithmic value of λ in the horizontal coordinate, the error value in the vertical coordinate, and the number of predictive variables for different values of λ in the upper part of the figure, and the two dashed lines indicate lambda.min and lambda.1se, respectively. The model chose lambda.1se corresponding to a λ value of 0.03027783 and selected three predictors (hypertension, CVD, duration). The importance of each variable in predicting DR can be assessed using the odds ratios provided by multivariate logistic regression model (**Table 3**).

**Table 3:** Results of multivariate logistic regression model.

Variables	β	SE	OR(95%CI)	P value
Hypertension	0.969	0.116	2.635(2.099,3.308)	<0.001
CVD	0.435	0.141	1.545(1.172,2.037)	0.002
Duration	0.070	0.007	1.073(1.058,1.087)	<0.001

CVD: cardiovascular disease; SE: Standard Error; OR: odds ratio; CI: Confidence Interval.

### 3.3 Nomogram construction and performance

**Figure 2:**
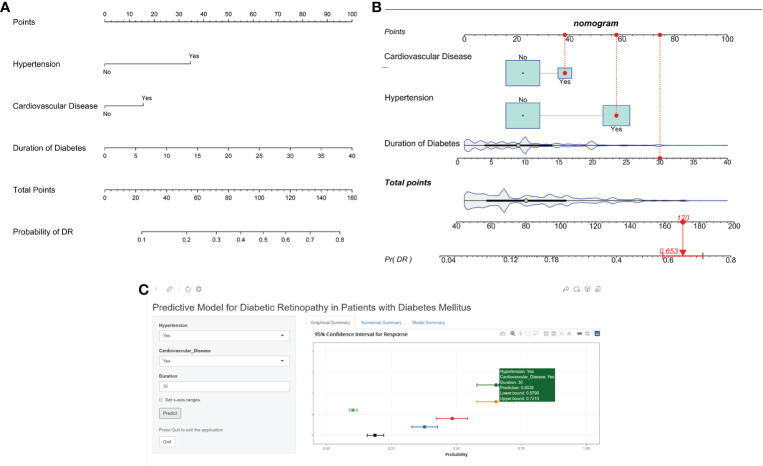
Nomogram identifying retinopathy in patients with DM. **(A)** Risk nomogram for identifying retinopathy in patients with DM. **(B)** One patient from our study is shown as an example (presented in red). **(C)** An example of Nomogram to identifying retinopathy in patients with DM Via a Link.

To visualize the diagnostic model, we developed a nomogram that provides a convenient and personalized presentation tool for predicting the probability of DR (**Figure 2A**). Points were assigned for all risk factors, first by drawing a line upward from the corresponding value to the “Points” line to get the points for each factor, then the points for all factors were added to obtain the total points and a vertical line was drawn to the “Total point” row to determine DR occurrence. One patient from our study was shown as an example (presented in red). The distinct area of rectangles represented the difference of the relative proportion of patients in each subgroup (**Figure 2B**).

**Figure 3:**
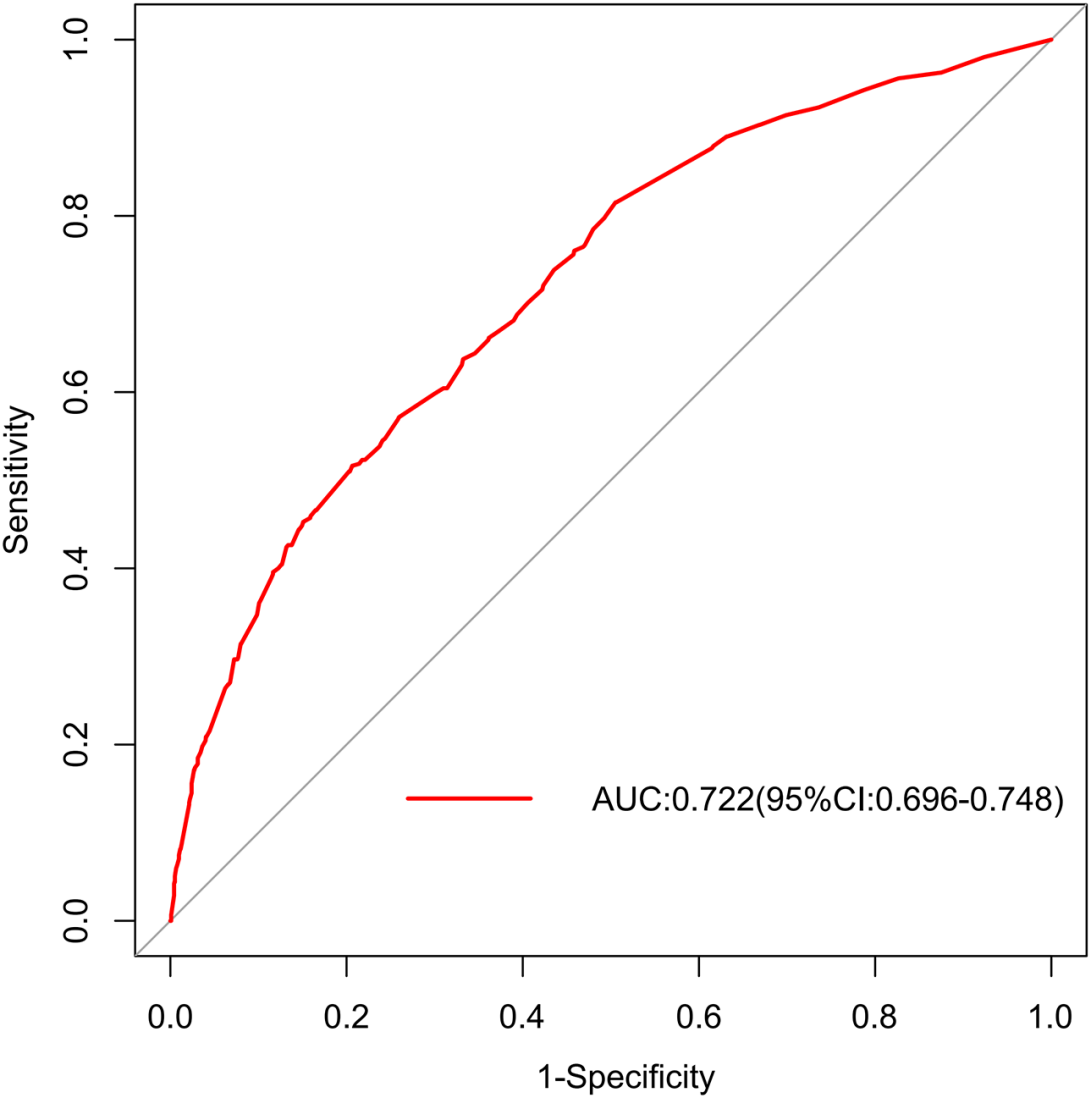
AUROC in training set.

**Figure 4:**
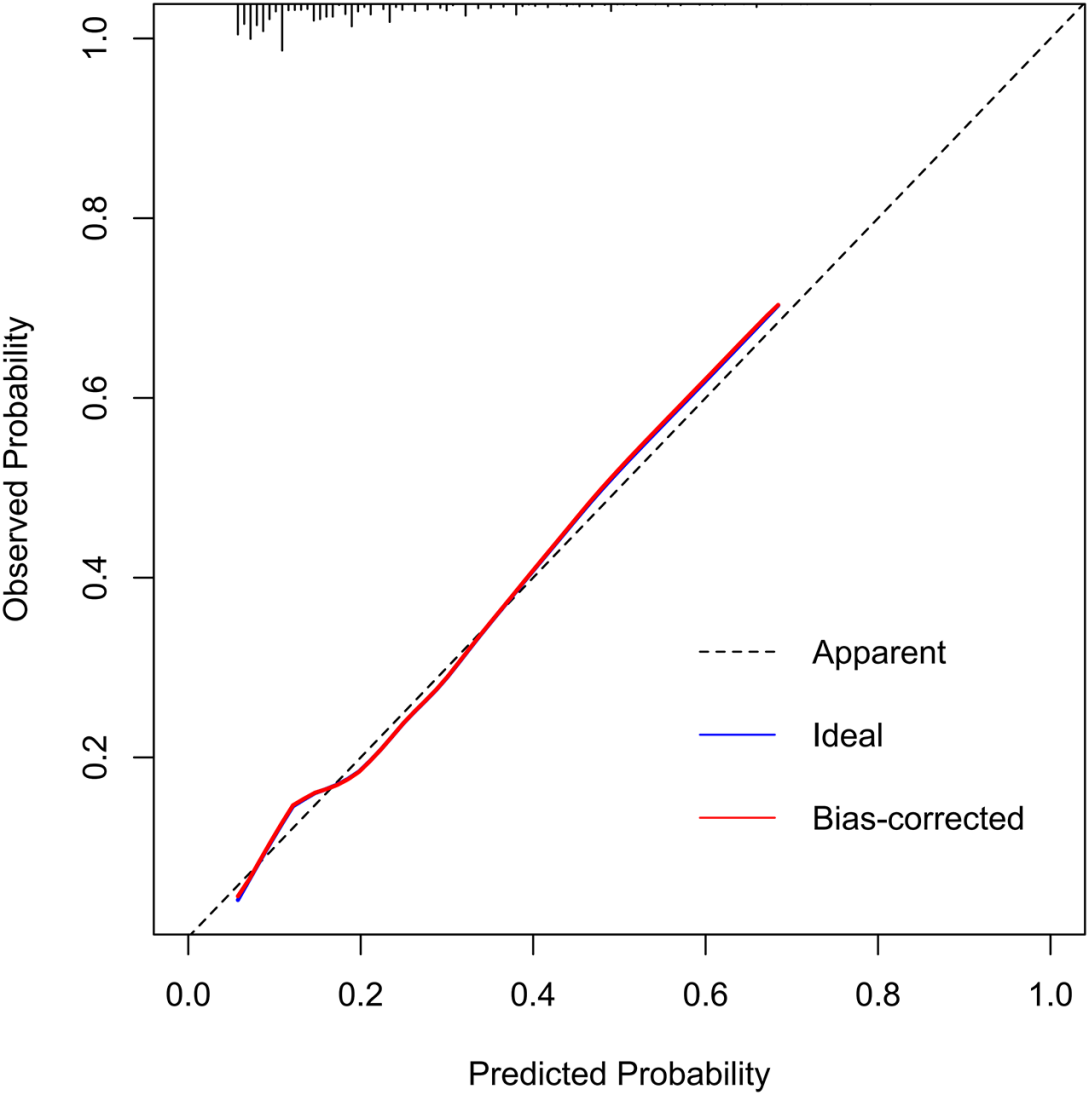
Calibration curve of the DR incidence risk nomogram prediction in training set.

In the training set, the area under the AUROC curve was 0.722 (95% CI:0.696-0.748), which showed that the diagnostic model had good discriminatory ability (**Figure 3**). The calibration curve (bootstraps=1000) suggested that the predicted probability was highly consistent with the actual probability (**Figure 4**). The AUROC curves of the nomogram were 0.712 (95% CI:0.670-0.754) and 0.703 (95% CI:0.552-0.853) in the internal validation and external validation sets, respectively (**Supplementary Figures S2**, **S3**). **Table 4** lists the detailed performance metrics of the three datasets.

**Table 4:** Detailed performance metrics for the three datasets.

Models	AUC	Sensitivity	Specificity	PPV	NPV
	(95%CI)	(95%CI)	(95%CI)	(95%CI)	(95%CI)
Training set	0.722	0.571	0.741	0.317	0.891
	0.696-0.748	0.526-0.617	0.722-0.79	0.286-0.349	0.877-0.906
Internal validation set	0.712	0.509	0.806	0.319	0.902
	0.670-0.754	0.434-0.584	0.781-0.831	0.263-0.374	0.882-0.922
External validation set	0.703	0.571	0.941	0.333	0.977
	0.553-0.853	0.360-0.783	0.919-0.964	0.179-0.487	0.962-0.992

AUC, area under the curve; PPV, positive predictive value; NPV, negative predictive value; CI, Confidence Interval.

Based on the original model, we combined other risk factors previously reported in the literature, including SBP, HbA1c, LDL-C, and all three. The performance of these models were compared using Delong test (**Table 5**). Logistic regression trained to detect DR in our model had an AUC of 0.722 (95% CI 0.696-0.748), non-inferior to the original model combined with SBP (AUC 0.723, 95% CI 0.698-0.749), LDL-C (AUC 0.726, 95% CI 0.700-0.752), HbA1c (AUC 0.721, 95% CI 0.695-0.747), and all three variables (AUC 0.726, 95% CI 0.700-0.752) (P=0.605, 0.247, 0.107, 0.286, respectively).

**Table 5:** Model performance statistics in training dataset.

Models	AUC	Sensitivity	Specificity	PPV	NPV	P value
	(95%CI)	(95%CI)	(95%CI)	(95%CI)	(95%CI)	
Model	0.722	0.571	0.741	0.317	0.891	/
	0.696-0.748	0.526-0.617	0.722-0.790	0.286-0.349	0.877-0.906	
Model+SBP	0.723	0.670	0.643	0.284	0.902	0.605
	0.698-0.749	0.627-0.714	0.622-0.663	0.257-0.311	0.887-0.917	
Model+LDL-C	0.726	0.765	0.557	0.267	0.918	0.247
	0.700-0.752	0.726-0.804	0.536-0.578	0.243-0.291	0.903-0.933	
Model+HbA1c	0.721	0.563	0.752	0.324	0.891	0.107
	0.695-0.747	0.517-0.608	0.734-0.770	0.291-0.356	0.876-0.905	
Model+SBP+HbA1c+LDL-C	0.726	0.776	0.550	0.267	0.921	0.286
	0.700-0.752	0.738-0.814	0.529-0.571	0.243-0.291	0.906-0.936	

SBP, systolic blood pressure; LDL-C, low-density lipoprotein cholesterol; HbA1c, glycated hemoglobin; AUC, area under the curve; PPV, positive predictive value; NPV, negative predictive value; CI, Confidence Interval. Symbol "/" means that "Model" is used as the reference object for AUC comparison.

### 3.4 Clinical utility of the nomogram

**Figure 5:**
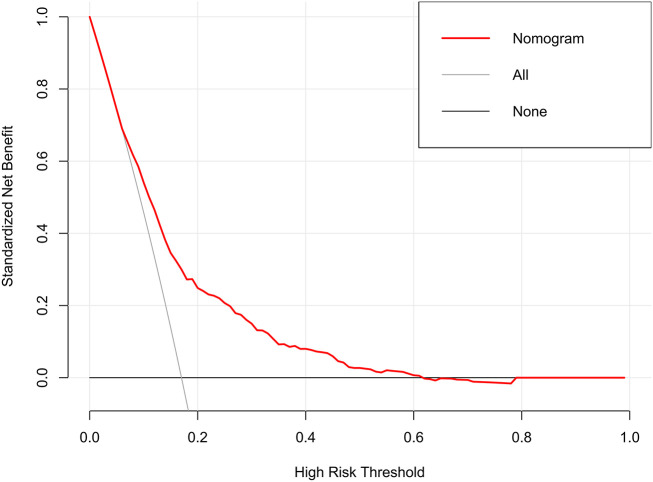
Decision curve analysis of the nomogram.

**Figure 6:**
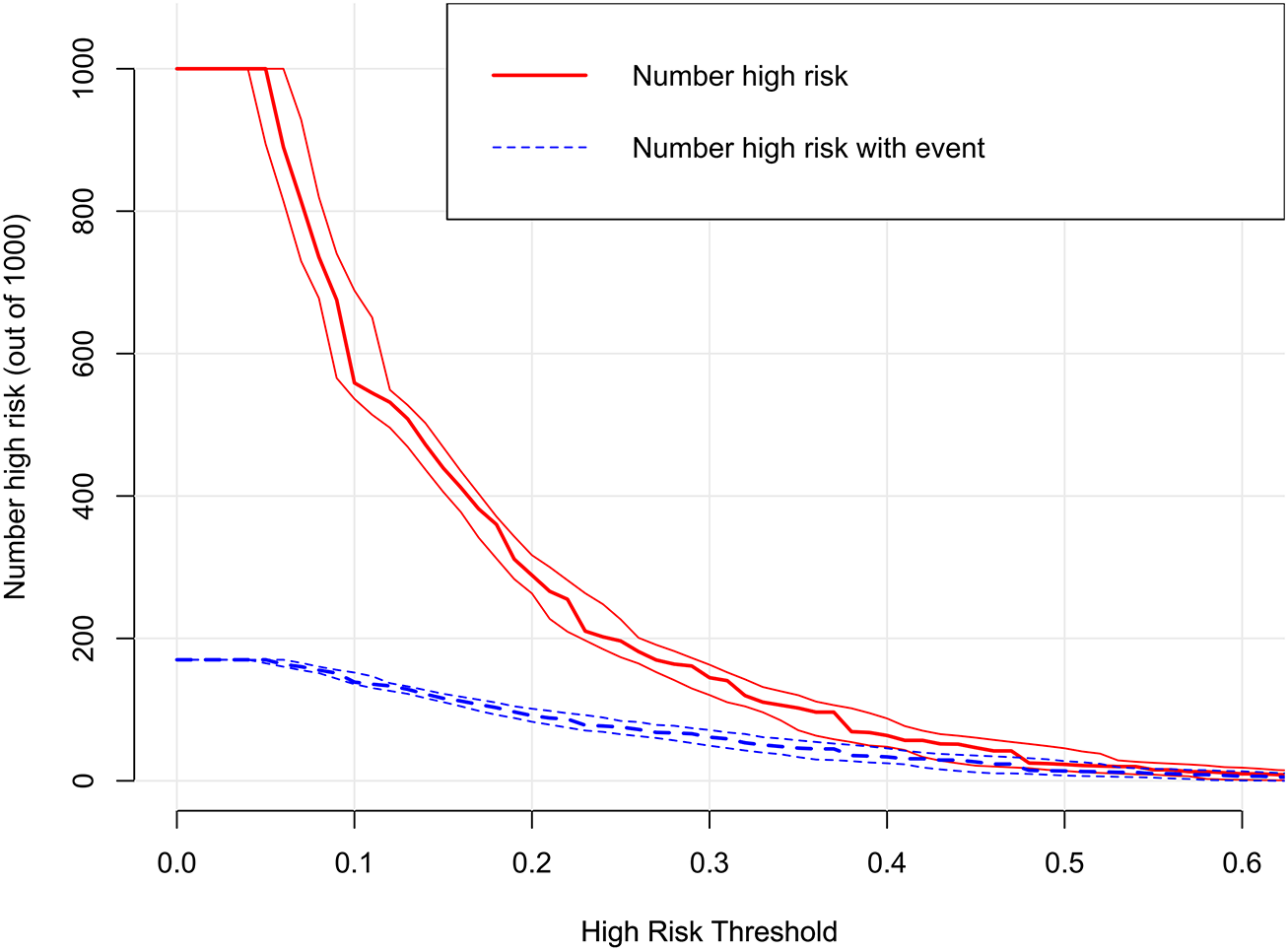
Clinical impact curve of the nomogram.

The decision curves showed that the threshold probability of DR in diabetic patients was 17-55% according to the nomogram (**Figure 5**), and that applying this nomogram to identify DR would provide greater benefit than an all-treatment regimen or an all-no-treatment regimen. In addition, further clinical impact curves were created to assess the nomogram’s clinical impact and provide a more intuitive understanding of its significance (**Figure 6**). The clinical impact curves depicted the estimated number of diabetic patients with DR at each risk threshold as well as the actual number of patients presenting with DR. When the risk threshold exceeded 30%, the estimated number of patients was closer to the actual number of patients.

### 3.5 Construction of web app to easily access the nomogram

Finally, we developed a user-friendly interface on a web link (https://cqmuxss.shinyapps.io/dr_tjj/) to calculate the precise probability of concomitant DR in patients with diabetes. For the same example in **Figure 2B**, the likelihood of DR was 0.653 (95% CI:0.579-0.721) when a patient had both hypertension and CAD and the duration of diabetes was 30 years (**Figure 2C**).

## 4 Discussion

We found that 21.7% (455/2610) of patients had some degree of DR, and this percentage was in consistent with the report by Song (8), who described an 18.7% prevalence in patients with a similar duration and also from southwest China. Given that the current study comprised a hospitalized-based population with relatively severe conditions, including long-term diabetes (median duration, 9 years) and poor management of blood glucose (median glycosylated hemoglobin, 8.9%) on admission, the high prevalence of DR revealed by our data is not surprising.

Our study demonstrated that an easy-to-use diagnostic model can identify underlying DR with a high NPV (97.7% on an external validation dataset), high specificity (94.1%), and qualified sensitivity (57.1%). When the common predictors (HbA1c, lipids, and SBP) proposed in previous studies were considered, our original model’s output remained non-inferior to that of complex equations with a greater number of risk variables.

Previous studies reported that common risk factors for DR included both duration of DM and age at visit (10, 11); however, designing a prompt referral for DR screening should take into account the length of DM as a crucial determinant (4, 13). A longer duration may represent a longer period of hyperglycemia-induced retinal toxicity, which is believed to be associated with both vascular and neural retinal death. Recent studies have shown that there is a significant interaction between the patient’s age and the duration of DM, based on sensitivity analysis (11). In contrast, age was observed to be a protective factor for DR due to a state of low retinal perfusion in elderly patients (19). A nationwide population-based cohort study also showed a reduced prevalence of DR in individuals with diabetes of less than 5 years duration, and regular screening had no impact on detection rates among young patients (< 45 years) (34). Patients with a younger age of onset have a longer duration of diabetes, and there is an interaction between the patient’s age at visit, age at onset, and duration of diabetes. Consequently, studying the effect of one of these factors on DR requires adjusting for the other two variables. Our findings suggest that age was only subordinate in determining the prevalence of DR, compared to the duration of diabetes, which was a predominant predictor.

Earlier studies revealed that glycated hemoglobin (10, 11, 13, 15–17, 19) or glycemic variability (20, 21) could be predictors for DR, but structured glycemic detection adherence was highly correlated with social support (24). Local experience-based studies from China, India, and Brazil have indicated that the routine use of SMBG was frequently challenging, mainly because of the out-of-pocket expenditures associated with glucose monitoring (35). In comparison to our simple model, mandatory inclusion of hemoglobin A1c did not substantially increase the AUROC. A previous study suggested that glucose variability was linked to the development and progression of DR (20), but no significant relationships were found after adjusting for hemoglobin A1c (36), making this association less relevant. Glycemic records from continuous glucose monitoring systems were not collected in our study, as this novel technology is not available in most cases with limited medical resources.

The relationship between elevated blood lipid profiles and DR development of DR was complex. Previous studies on the correlation between conventional serum lipid levels (TG, TC, HDL-C, and LDL-C) and DR have shown conflicting results (10, 13, 19, 37). Similarly, the current focus on the role of lipoprotein(a) in the pathogenesis of DR remains controversial (38, 39).A recent study focused on the relationship between apolipoproteins and DR (40); however, these novel laboratory parameters have not yet been widely used in clinical practice. Our data support previous evidence that customary serum lipid levels were not strongly or consistently related to DR. Traditional lipid measures are unstable laboratory markers, and their serum levels are significantly altered by diets and lipid-lowering agents, which might be a possible explanation for this finding.

Increased blood pressure can lead to additional damage to the retinal vessels by hyperperfusion, shearing forces, and increased edema formation. Hypertension, mainly SBP, was previously identified as the most common modifiable risk factor for DR (4, 16); however, these variables did not present a steady correlation in other studies (13, 19). The association between poorly controlled hypertension and DR has not recently been observed in the Chinese population compared to this relationship in Malays and Indians (41). SBP was found to be a risk factor for DR in univariate analysis but was not included in the LASSO model in our study. A cross tabulation was created to assess whether antihypertensive drugs affected DR onset, with the chi-square test result revealing the independent of the two variables (P = 0.361, **Supplementary Table S2**), even though SBP was slightly lower in the medicated group than in the non-medicated group (141mmHg and 146mmHg, respectively, P = 0.026). Extensive background treatment with antihypertensive drugs in diabetic patients (86.4%, 868/1005, **Supplementary Table S2**) may remarkably alter blood pressure values within a single visit. On the other hand, SBP was 132mmHg, 135mmHg, 138mmHg in the short-duration (< 5 years), medium-duration (5-10 years), and long-duration (≥10 years) group, respectively (F=19.387, P < 0.001, **Supplementary Table S3**). Therefore, the length of diabetes might be a potential confounding variable in the SBP-DR correlation observed in univariate analysis, and comorbid hypertension was identified as a predictor of high importance.

Several studies have shown a correlation between DR and CVD, but most have concluded that DR is a predictor of CVD (42, 43). Multivariate logistic regression results based on the type 1 diabetes population revealed that patients with DR were more likely to develop CVD (42). Similarly, diabetic retinal vascular disease was recently found to predict CVD morbidity and mortality in patients with type 2 diabetes (43). In addition, according to univariate analysis, diabetic patients with a history of CVD had a 60% greater chance of developing sight-threatening DR than those without a history of CVD (11). Our study results are in accordance with those of previous reports. Although further research remains to be conducted to properly comprehend the relationship between DR and CVD, our findings confirmed the continuum of diabetic vascular disease, showing that microangiopathy and macroangiopathy appeared to be interrelated rather than distinct conditions. Another important implication is that comorbid CVD may prompt PHC to identify a subset of patients with diabetes for priority eye examinations.

Some antidiabetic drugs are thought to be associated with an increased risk of DR. TZD can cause fluid retention and peripheral edema in patients with diabetes (5), and systematic fluid retention may emerge as diabetic macular edema. However, the ACCORD Eye Study group reported no consistent evidence of DR progression in patients receiving TZD, suggesting that this correlation requires further study (44). For GLP-1 RAs, worsening of retinopathy has been considered a new potential side effect of this treatment. An unexpected increase in retinopathy was found in SUSTAIN-6, and a nonsignificantly higher rate of DR was found in LEADER and REWIND (45). However, poor outcomes, such as blindness or the need for numerous procedures, which damage a person’s quality of life and raise expenditures, were not adequately described, and definitions of ocular events varied widely among these studies. The first answer to these issues may come from a trial that is currently underway (FOCUS trial; NCT03811561), which will examine the long-term impact of semaglutide on diabetic eye complications (up to 5 years). Data from our study showed that TZD and DPP-4i were risk factors, whereas GLP-1 RAs were protective factors in the univariate analysis. However, all of them failed to enter the LASSO regression, not ruling out a false-positive result due to the low usage rate of these three drugs (4.48%, 2.18%, and 2.84%, respectively). AGI or Met alone cannot usually manage blood glucose in diabetic patients with a median length of 9 years, and positive results in univariate analysis could include the effect of other drugs. The use of insulin tended to be highly correlated with duration, which may explain why the risk effect was not found in the LASSO regression.

In 2020, the annual per capita disposable income of rural households in China was approximately 17,132 yuan, which is approximately one-third of the income of urban households (25). Previous findings revealed that only 10% of DR patients in rural China were properly diagnosed and treated, nearly 70% of DM subjects had never received an eye exam (46), and over 70% of participants considered financial cost as the leading barrier to routine retinal screening (47). Significant variations in the development and severity of DR have been found in clinical studies of patients with DM, but the variations have not been fully elucidated by known risk factors (23). Thus, it is important to minimize the number of variables in diagnostic tools as much as possible in medically underserved settings. The population with limited access to ophthalmologic care may benefit from our diagnostic model, which was developed based on restricted medical resources and would not incur additional expenditures. The easy-to-use web calculator will help PHC practitioners quickly identify at-risk individuals for DR and make prompt referrals.

The strengths of the current study include the use of a large sample from multicenter electronic medical records of diabetic individuals with or without DR. However, our study was subject to some limitations. First, our study had a cross-sectional design. Compared to cohort studies, cross-sectional studies provide weaker evidence, and the interpretation of these findings should be considered with caution. Second, the current dataset had an incomplete recording of UACR with 58.74% missing values and HbA1c with 14.6% missing values. We could not account for the effect of albuminuria on DR, even though multiple imputations were used to address missing HbA1c values. Therefore, further studies with complete data for all pertinent covariates would be useful. In addition, the efficacy of our model for indetifying referral DR needs to be validated in community patients, but this requires complete medical records both in the community and the hospital. Finally, it is not clear whether our findings based on patients with type 2 diabetes (2584/2610) are applicable to patients with type 1 diabetes, while few data on type 1 diabetes suggest that the retinal risk factors were generally similar (16).

## 5 Conclusions

Our study suggests that a simple predictive model could provide added value as an automated screening tool to triage patients for priority retinal examination. Obtaining information on the duration of diabetes, history of hypertension, and CVD requires no additional medical costs and is convenient. Further validation studies on the proposed model are required. Moreover, this model cannot replace standard DR screening but could be more reasonable as a timely warning tool, and a more effective option is to promote a nationwide DR examination program for all diabetic patients.

## Data availability statement

The raw data supporting the conclusions of this article will be made available by the authors, without undue reservation.

## Ethics statement

The Ethics Committee of the Affiliated Banan Hospital of Chongqing Medical University approved the study. Written informed consent for participation was not required for this study due to its retrospective design, and the study was undertaken in accordance with national legislation and institutional requirements.

## Author contributions

YY and JT designed the research. YY, JT, HH, JG, and YL collected and organized data. YY, JT, YH, and TW analyzed the data. YY and JT drafted the manuscript. QZ and XX contributed to the critical revision of the manuscript. All authors contributed to the manuscript and approved the submitted version.
