# Supplementary material for: Predictive model for diabetic retinopathy under limited medical resources: A multicenter diagnostic study
Source: Front Endocrinol (Lausanne). 2023 Jan 5;13:1099302. doi: 10.3389/fendo.2022.1099302 (PMC9849672; doi:10.3389/fendo.2022.1099302)
Supplement: Supplementary file 1 [file DataSheet_1.docx]

**Supplementary information**

**Figure S1. Flow of inclusions and exclusions**

**
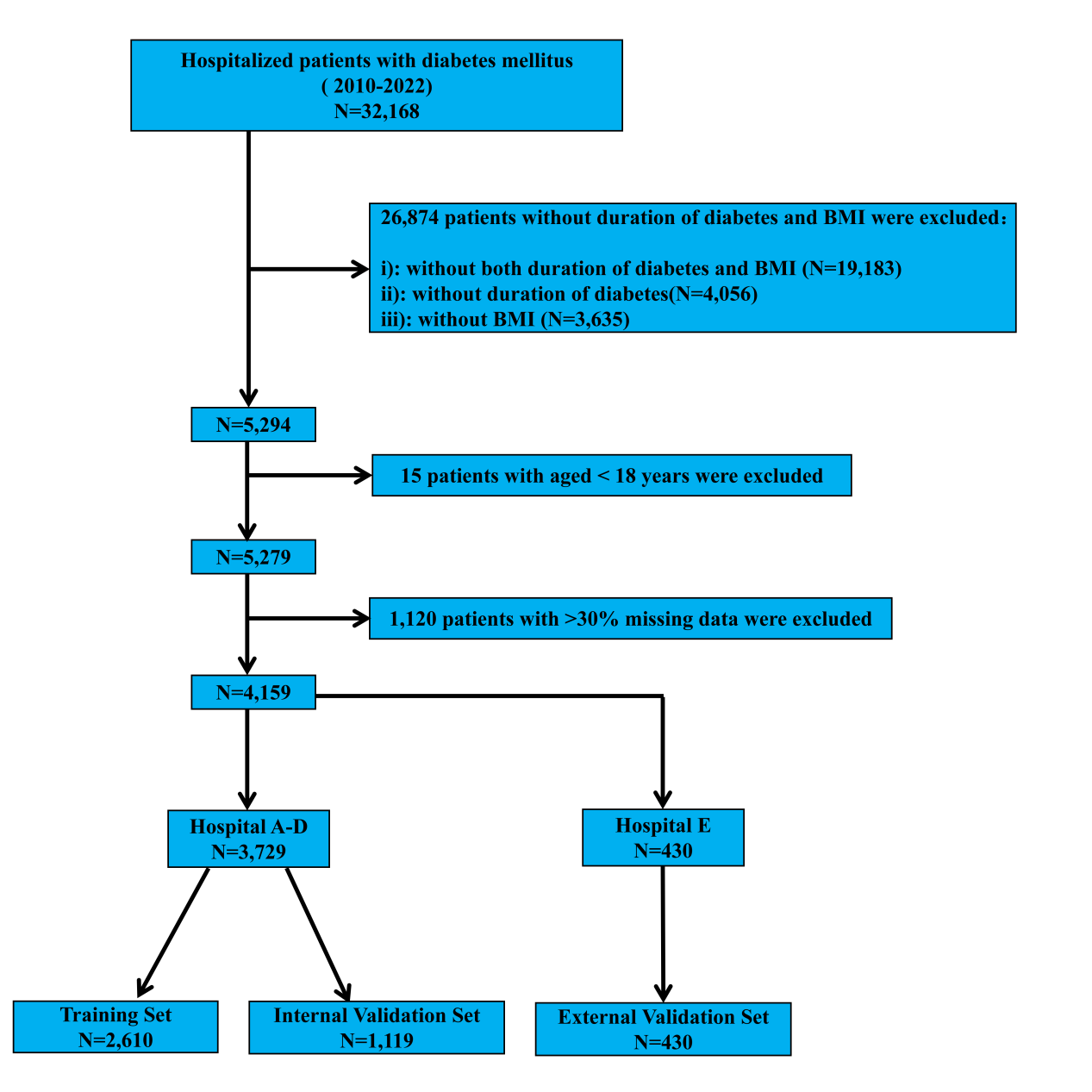
**

**Table S1. ICD-10 codes related to hypertension, CVD and DR for our study**

| **Code** | **Description** |
| --- | --- |
| **Hypertension** | |
| I10.x00 | Essential (primary) hypertension |
| I11.000 | Hypertensive heart disease with (congestive) heart failure |
| I11.900 | Hypertensive heart disease without (congestive) heart failure |
| I12.000 | Hypertensive renal disease with renal failure |
| I12.900 | Hypertensive renal disease without renal failure |
| I13.000 | Hypertensive heart and renal disease with (congestive) heart failure |
| I13.100 | Hypertensive heart and renal disease with renal failure |
| I13.200 | Hypertensive heart and renal disease with both (congestive) heart failure and renal failure |
| **Diabetic Retinopathy** | |
| E10.3 | Type 1 diabetes mellitus with ophthalmic complications |
| E10.31 | Type 1 diabetes mellitus with unspecified diabetic retinopathy |
| E10.311 | Type 1 diabetes mellitus with unspecified diabetic retinopathy with macular edema |
| E10.319 | Type 1 diabetes mellitus with unspecified diabetic retinopathy without macular edema |
| E10.32 | Type 1 diabetes mellitus with mild nonproliferative diabetic retinopathy |
| E10.321 | Type 1 diabetes mellitus with mild nonproliferative diabetic retinopathy with macular edema |
| E10.329 | Type 1 diabetes mellitus with mild nonproliferative diabetic retinopathy without macular edema |
| E10.33 | Type 1 diabetes mellitus with moderate nonproliferative diabetic retinopathy |
| E10.331 | Type 1 diabetes mellitus with moderate nonproliferative diabetic retinopathy with macular edema |
| E10.339 | Type 1 diabetes mellitus with moderate nonproliferative diabetic retinopathy without macular edema |
| E10.34 | Type 1 diabetes mellitus with severe nonproliferative diabetic retinopathy |
| E10.341 | Type 1 diabetes mellitus with severe nonproliferative diabetic retinopathy with macular edema |
| E10.349 | Type 1 diabetes mellitus with severe nonproliferative diabetic retinopathy without macular edema |
| E10.35 | Type 1 diabetes mellitus with proliferative diabetic retinopathy |
| E10.351 | Type 1 diabetes mellitus with proliferative diabetic retinopathy with macular edema |
| E10.359 | Type 1 diabetes mellitus with proliferative diabetic retinopathy without macular edema |
| E10.39 | Type 1 diabetes mellitus with other diabetic ophthalmic complication |
| E11.3 | Type 2 diabetes mellitus with ophthalmic complications |
| E11.31 | Type 2 diabetes mellitus with unspecified diabetic retinopathy |
| E11.311 | Type 2 diabetes mellitus with unspecified diabetic retinopathy with macular edema |
| E11.319 | Type 2 diabetes mellitus with unspecified diabetic retinopathy without macular edema |
| E11.32 | Type 2 diabetes mellitus with mild nonproliferative diabetic retinopathy |
| E11.321 | Type 2 diabetes mellitus with mild nonproliferative diabetic retinopathy with macular edema |
| E11.329 | Type 2 diabetes mellitus with mild nonproliferative diabetic retinopathy without macular edema |
| E11.33 | Type 2 diabetes mellitus with moderate nonproliferative diabetic retinopathy |
| E11.331 | Type 2 diabetes mellitus with moderate nonproliferative diabetic retinopathy with macular edema |
| E11.339 | Type 2 diabetes mellitus with moderate nonproliferative diabetic retinopathy without macular edema |
| E11.34 | Type 2 diabetes mellitus with severe nonproliferative diabetic retinopathy |
| E11.341 | Type 2 diabetes mellitus with severe nonproliferative diabetic retinopathy with macular edema |
| E11.349 | Type 2 diabetes mellitus with severe nonproliferative diabetic retinopathy without macular edema |
| E11.35 | Type 2 diabetes mellitus with proliferative diabetic retinopathy |
| E11.351 | Type 2 diabetes mellitus with proliferative diabetic retinopathy with macular edema |
| E11.359 | Type 2 diabetes mellitus with proliferative diabetic retinopathy without macular edema |
| E11.39 | Type 2 diabetes mellitus with other diabetic ophthalmic complication |
| **Cardiovascular Disease** | |
| I25 | Chronic ischemic heart disease |
| I25.1 | Atherosclerotic heart disease of native coronary artery |
| I25.10 | Atherosclerotic heart disease of native coronary artery without angina pectoris |
| I25.11 | Atherosclerotic heart disease of native coronary artery with angina pectoris |
| I25.110 | Atherosclerotic heart disease of native coronary artery with unstable angina pectoris |
| I25.111 | Atherosclerotic heart disease of native coronary artery with angina pectoris with documented spasm |
| I25.118 | Atherosclerotic heart disease of native coronary artery with other forms of angina pectoris |
| I25.119 | Atherosclerotic heart disease of native coronary artery with unspecified angina pectoris |
| I25.2 | Old myocardial infarction |
| Z95.1 | Presence of aortocoronary bypass graft |
| Z95.5 | Presence of coronary angioplasty implant and graft |

**Table S2. Diabetic patients coexist with hypertension stratified by retinopathy status and antihypertensive treatment**

|  | DR group | Non-DR group | Total | P value |
| --- | --- | --- | --- | --- |
| with antihypertensive drug | 252 | 616 | 868 | 0.361 |
| without antihypertensive drug | 34 | 103 | 137 |  |
| Total | 286 | 719 | 1005 |  |

*DR: diabetic retinopathy.*

**Table S3. Effects of diabetic duration on the difference of SBP**

| Duration | N | SBP (IQR, mmHg) | F | P value |
| --- | --- | --- | --- | --- |
| short-duration (< 5 years) | 718 | 132.00(120.00,146.00) | 19.387 | <0.001 |
| medium-duration (5-10 years) | 627 | 135.00(122.00,148.00) |  |  |
| long-duration (≥10 years) | 1265 | 138.00(126.00,150.00) |  |  |

*SBP: systolic blood pressure; IQR: interquartile range.*

**Figure S2. AUC of the ROC curve in internal validation set**

**
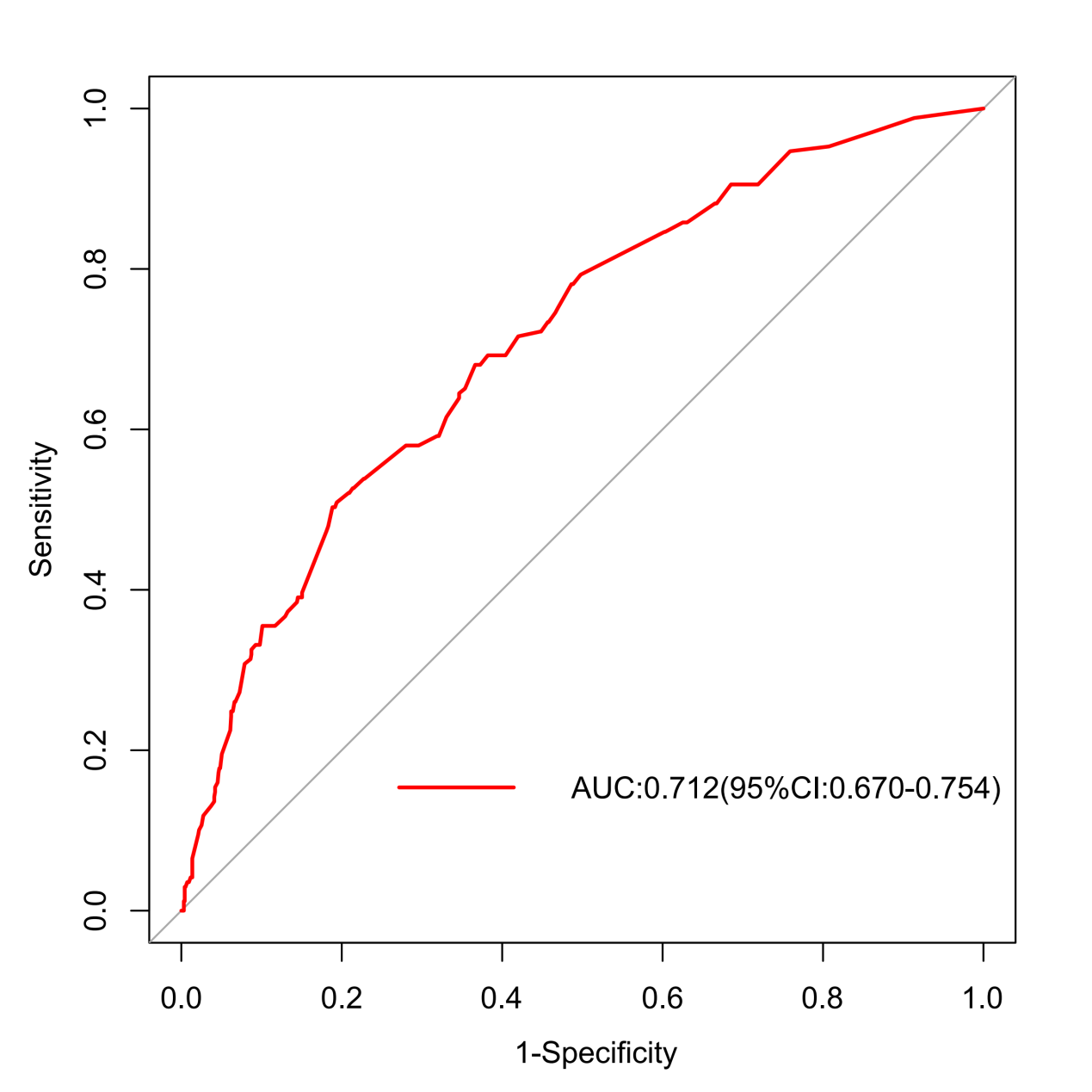
**

**Figure S3. AUC of the ROC curve in external validation set**

**
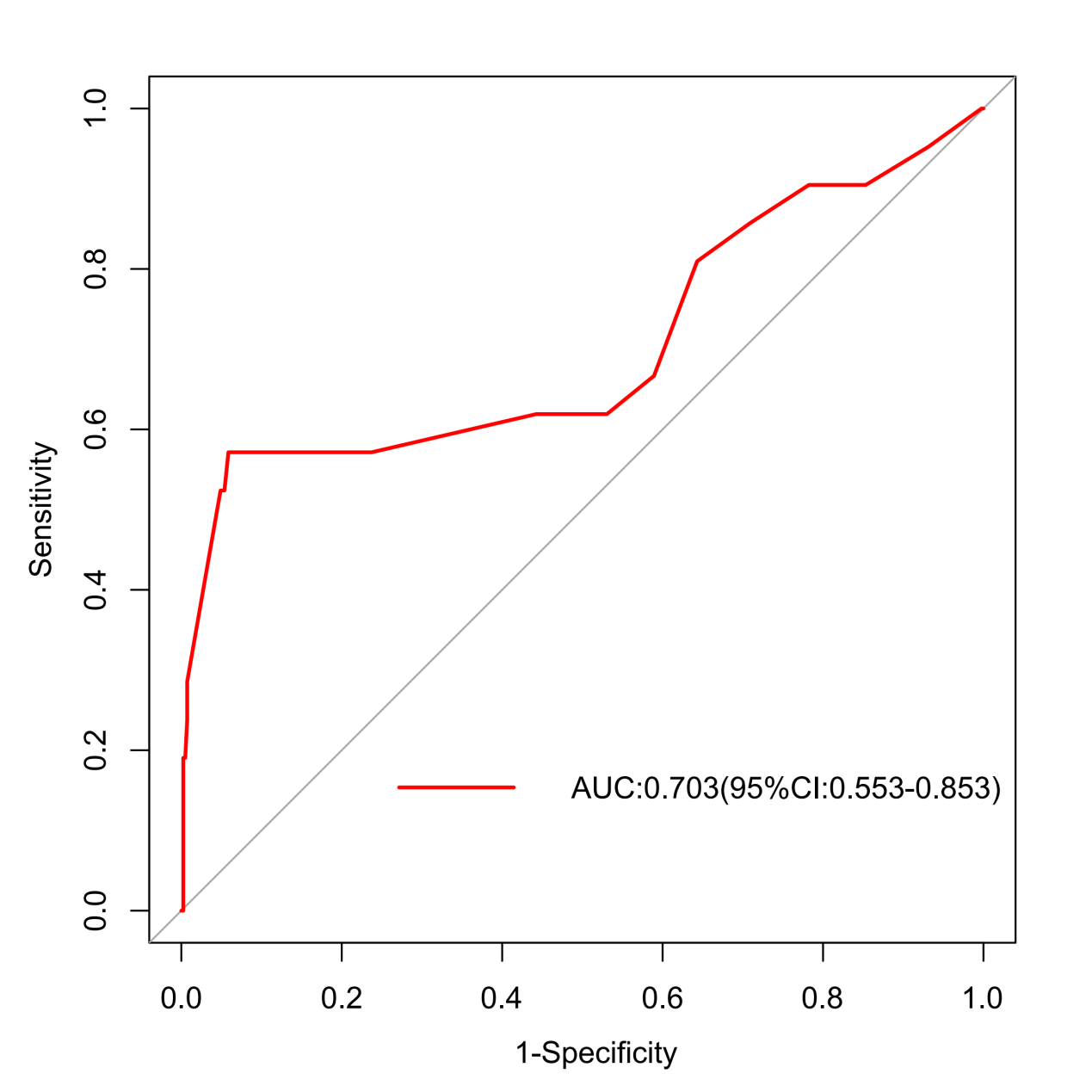
**
